# A Novel Invasive Hemodynamic Parameter (V′) to Predict Hemodynamic Response to Mitral Transcatheter Edge-to-Edge Repair

**DOI:** 10.1016/j.shj.2026.101069

**Published:** 2026-06-09

**Authors:** Maryam Muhammad Ali Majeed-Saidan, Shivabalan Kathavarayan Ramu, Besir Besir, Odette Iskandar, James Yun, Rhonda Miyasaka, Amar Krishnaswamy, Serge Harb, Samir R. Kapadia

**Affiliations:** Heart, Vascular and Thoracic Institute, Cleveland Clinic, Cleveland, Ohio, USA

**Keywords:** LA mean pressure, LA V wave, Mitral regurgitation, Mitral transcatheter edge-to-edge repair, Mitral valve, V prime

## Abstract

**Background:**

Left atrial (LA) V-wave amplitude can be higher with elevated left ventricular end-diastolic pressure, mitral regurgitation (MR), or both. This may cause hemodynamic and echocardiographic discordance when assessing MR. Identifying more specific hemodynamic correlates to MR severity helps in the setting of discordance. We examined the amplitude of the V-wave relative to the LA mean pressure following the reduction in MR after mitral transcatheter edge-to-edge repair (M-TEER).

**Methods:**

A total of 231 patients who underwent M-TEER between 2019 and 2022 with reliable intraprocedural invasive hemodynamic waveforms were included. LA mean and V-wave pressures were measured pre- and post-M-TEER. V′ was calculated as follows: (V′ = V-wave – mean pressure).

**Results:**

Our cohort (median age 79) included 65% and 35% patients with primary mitral regurgitation (PMR) and secondary mitral regurgitation (SMR), respectively. LA mean, V-wave, and V′ pressures decreased significantly after M-TEER in both groups. SMR patients had higher LA pressures after M-TEER. Baseline and post–M-TEER V′ did not differ among groups. In the overall cohort, pre– and post–M-TEER V′ were significantly higher in patients with >2+ residual MR compared to ≤2+ MR. Patients with post–M-TEER V′ >13 mmHg had worse composite outcomes of heart failure hospitalization or all-cause mortality (hazard ratio: 3.01; 95% CI: 1.68-5.4; *p* < 0.001).

**Conclusion:**

M-TEER decreased LA mean, V-wave, and V′ pressures in PMR and SMR. Postprocedural V′ provides a useful measure of residual MR and clinical outcomes and guides the adequacy of M-TEER.

## Highlights

• Changes in LA mean and V-wave pressures after mitral transcatheter edge-to-edge repair (M-TEER) have been previously studied and established in the literature.
• V′ was found to be a better correlate of residual mitral regurgitation independent of left ventricular filling pressures.
• The V′ is a readily available intraprocedural parameter that can be used to guide the M-TEER procedure, where less than 13 mm Hg V′ was associated with better clinical outcomes.

## Graphical Abstract

Graphic Abstract: Invasive LA Pressure Waveform Demonstrating Change in LA Mean, V-Wave, and V′ Pressures After M-TEER in PMR and SMR. This illustration depicts the invasive intraprocedural hemodynamic waveform of the left atrium pre- and post-M-TEER and shows the change in LA mean, V wave, and V′ pressures after the procedure in patients who had PMR and SMR. LA, left atrial; V′, V prime; M-TEER, mitral transcatheter edge-to-edge repair; PMR, primary mitral regurgitation; SMR, secondary mitral regurgitation; MR, mitral regurgitation.

**Figure:**
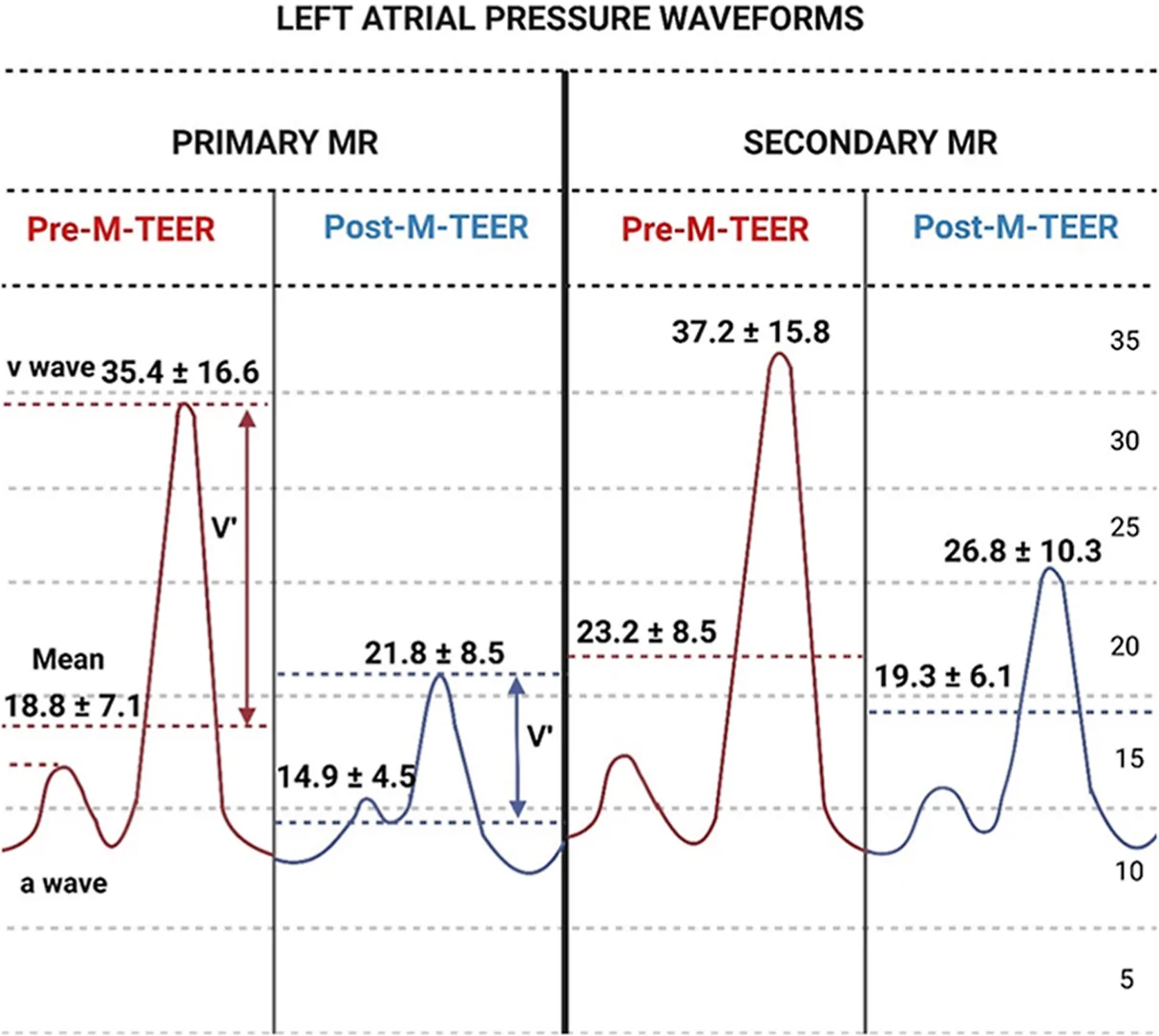

## Introduction

Mitral transcatheter edge-to-edge repair (M-TEER) is now an established therapy for patients with secondary mitral regurgitation (SMR) and surgically high-risk patients with primary mitral regurgitation (PMR). The success of the M-TEER procedure has been correlated to residual mitral regurgitation (MR) after the procedure.^1^^,^^2^ Residual MR is determined by echocardiographic assessment using analyses of the MR jet with color Doppler and flow patterns of the pulmonary vein (PV) and mitral valve (MV). Hemodynamic assessment may provide additional information to quantify residual MR and predict clinical outcomes. Post–M-TEER reductions in MR as determined by echocardiography are generally accompanied by decreases in the V wave. However, discrepancies are common, especially in the setting of elevated filling pressures. Such discordances pose intraprocedural challenges when deciding whether to deploy additional M-TEER devices. We hypothesized that these discrepancies arise from the fact that the V wave amplitude reflects both mean left atrial (LA) pressure and MR. We propose V prime (V′), a measure of the V wave peak from the mean LA pressure, may isolate the MR contribution to the V wave and that reductions in V′ may correlate better with reductions in MR severity and improved clinical outcomes.

## Methods

### Study Design

**Figure 1:**
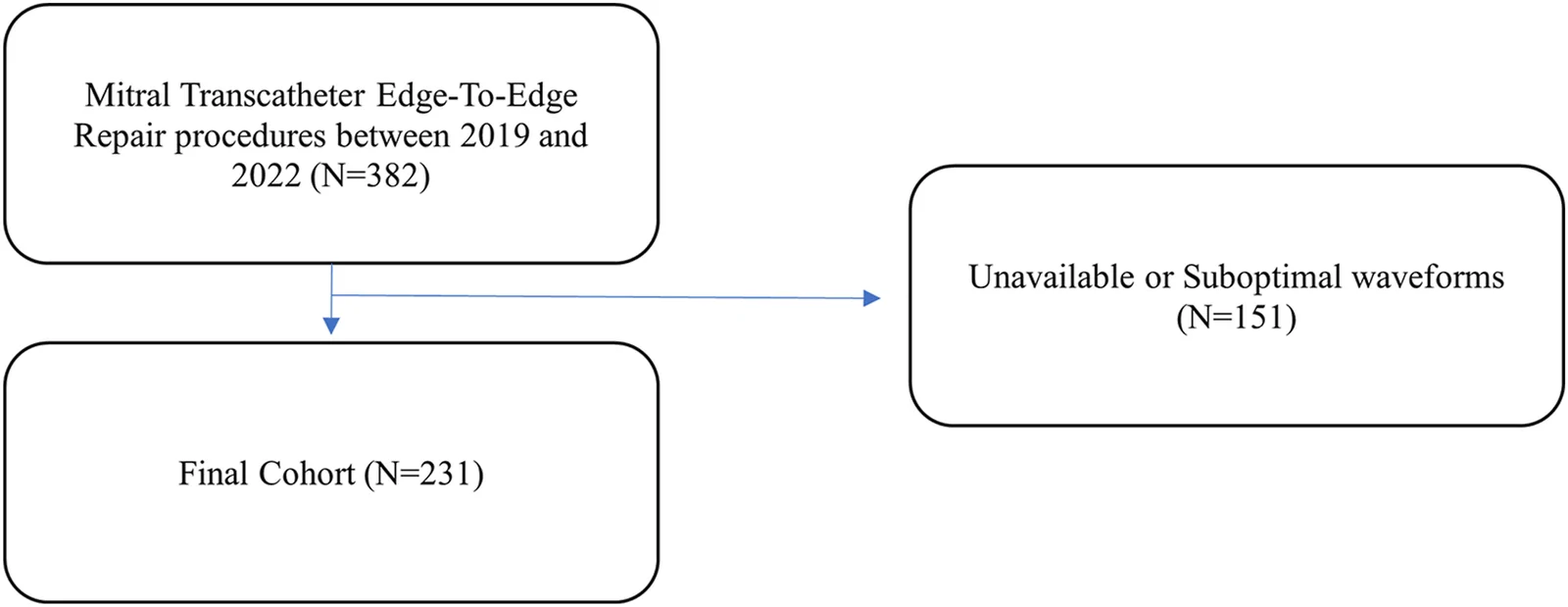
**Flow diagram illustrating the study patient population**.

We retrospectively identified 382 consecutive patients who underwent M-TEER for MR at the Cleveland Clinic, Cleveland, Ohio, between 2019 and 2022. We excluded 151 patients due to unavailable or suboptimal intraprocedural invasive hemodynamic waveforms, and the final cohort had 231 patients who underwent M-TEER in the study period (Figure 1). The study was approved by the institutional review board at our institution. A waiver of the requirement to obtain informed consent was obtained for the study.

### Outcomes

The primary outcome measure was the change in LA V-wave, mean pressure, and V′ among PMR and SMR patients. Procedural success was defined as a reduction in MR to ≤2+ postprocedure.^3^^,^^4^

## Measurement of Variables

### Baseline Characteristics

Baseline characteristics included age, sex, body mass index, New York Heart Association class, Society of Thoracic Surgeons (STS) MV repair risk score, and comorbidities such as hypertension, coronary artery disease, atrial fibrillation, end-stage renal disease, and diabetes mellitus.

### Invasive Hemodynamic Measurements

Invasive intraprocedural hemodynamic waves were measured and recorded during the procedure. They were then retrospectively reviewed using Syngo Dynamics software (Siemens Medical Solutions Inc, USA) to measure LA V-wave, LA mean pressure, aortic systolic and diastolic blood pressures, and heart rate before and after M-TEER. V′, the systolic change in LA pressure above the mean LA pressure, was calculated as the difference between peak LA V-wave and LA mean pressure (V′ = V-wave – mean pressure) (Graphic Abstract). LA mean pressure and V-wave amplitude were measured using calibrated fluid-filled catheters, zeroed at the mid-axillary line. Pressure tracings were recorded under stable hemodynamic conditions, and values were averaged over 3–5 consecutive cardiac cycles, with the exclusion of ectopic beats and artifacts.

### Echocardiographic Evaluation

All patients underwent a comprehensive echocardiographic assessment using commercially available ultrasonography systems. Echocardiographic parameters assessed in this study included left ventricular (LV) ejection fraction (LVEF), LV end-diastolic diameter, LV stroke volume, LA volume index, right ventricular systolic pressure, transvalvular mitral mean gradient, MV E wave, MV lateral and septal e’, and PV systolic (S) and diastolic (D) flow waves. Peak PV flow S and D wave velocities were measured from Doppler images recorded in the intraprocedural transesophageal echocardiogram before and after M-TEER. If the pulmonary venous flow pattern showed forward systolic flow with late systolic flow reversal, the peak velocity of the reversed systolic flow wave was measured and given a negative sign. All other echocardiographic parameters were assessed from preprocedural and postprocedural transthoracic echocardiography.

The patients were classified into 2 groups according to the cause of MR: patients with PMR and patients with SMR. Patients who had mixed-etiology MR were classified into PMR and SMR according to their predominant pathology.

### Clinical Outcomes

The clinical endpoints, namely all-cause mortality and heart failure hospitalization (HFH), were extracted from electronic health records.

## Statistical Analysis

Continuous variables are presented as mean ± SD and categorical variables as frequencies and percentages. Continuous variables were compared using a paired *t*-test and an independent sample *t*-test as appropriate. Categorical variables were compared using the chi-square test or Fisher exact test as appropriate. Pearson correlation was used to assess whether the invasive hemodynamic parameters were associated with echocardiographic parameters. Survival was assessed using a Kaplan–Meier curve and compared using the log-rank test. An unadjusted and adjusted Cox regression analysis was also performed to assess the difference in clinical outcomes among patients with higher vs. lower post–M-TEER V′s. The proportional hazard assumption was assessed using Schoenfeld residuals. A *p* value of <0.05 was considered statistically significant. SPSS ver. 23.0 (IBM Corp., USA) was used to perform chi-square tests, Fisher exact tests, independent sample *t*-tests, and paired *t*-tests. R Studio was used to execute Cox regression and survival analysis. The cut point of 22 was based on the median post-M-TEER V wave of the population.

We evaluated the association between post–M-TEER V′ and the 1-year composite outcome using a data-driven threshold approach. An optimal cutoff for post-M-TEER V′ was identified using maximally selected rank statistics (maxstat) based on the log-rank test. This method systematically tests all possible cut points and selects the threshold that maximizes separation in survival between groups. It was implemented using the maximally selected rank statistics algorithm (Lausen and Schumacher method) via the *survminer* and *maxstat* packages in R. Because this approach involves multiple testing across many potential cutoffs, we applied a Monte Carlo–based *p* value correction (conditional Monte Carlo method) to obtain a statistically valid significance level. Specifically, 1000 Monte Carlo simulations (B = 1000) were performed to estimate the distribution of the test statistic under the null hypothesis and adjust for the inherent multiple comparisons. To further assess the robustness of the identified cutoff, we performed bootstrap validation with 1000 resamples. In each bootstrap sample, the optimal cutoff was re-estimated using the same maxstat procedure. This allowed us to evaluate the stability of the cutoff across resampled datasets. The distribution of bootstrapped cutoffs yielded a 95% CI of (2-14), reflecting the variability and reliability of the threshold estimate.

## Results

### Baseline Characteristics

Out of 231 patients in our cohort, the median age of the study population was 79 (interquartile range: 72-85), 41% were females, and patients had an STS MV repair mean score of 7.87 ± 8.56. Patients with PMR were older (83 [77-87] vs. 73 [65-79], *p* < 0.001) and had lower body mass index (25.70 ± 5.19 vs. 28.59 ± 7.24, *p* = 0.001) and STS MV repair scores (6.53 ± 6.66 vs. 10.31 ± 10.87, *p* = 0.002) when compared to patients with SMR. The proportion of patients with diabetes mellitus was higher in SMR patients (31% vs. 17%; *p* = 0.02). Baseline New York Heart Association class differed significantly between the 2 groups (*p* = 0.02), but other baseline characteristics were comparable (Table 1).

**Table 1:** Baseline characteristics

	Overall (N = 231)	PMR (N = 149, 65%)	SMR (N = 82, 35%)	*p* value
Age	79 (72-85)	83 (77-87)	73 (65-79)	**<0.001**
Female (%)	95 (41)	63 (42)	32 (39)	0.63
BMI	26.72 ± 6.14	25.70 ± 5.19	28.59 ± 7.24	**0.001**
STS risk score MV repair	7.87 ± 8.56	6.53 ± 6.66	10.31 ± 10.87	**0.002**
Hypertension (%)	188 (81)	118 (79)	70 (85)	0.25
Diabetes mellitus (%)	51 (22)	26 (17)	25 (31)	**0.02**
CAD (%)	163 (71)	103 (69)	60 (73)	0.52
ESRD (%)	6 (3)	3 (2)	3 (4)	0.67
Atrial fibrillation (%)	152 (66)	93 (62)	59 (72)	0.14
Baseline NYHA				
II (%)	69 (30)	51 (40)	18 (28)	**0.02**
III (%)	111 (48)	73 (57)	38 (59)	
IV (%)	12 (5)	4 (3)	8 (13)	

Age is represented as median (IQR).

Categorical variables are represented as N (%).

Continuous variables are represented as mean ± SD.

Abbreviations: BMI, body mass index; CAD, coronary artery disease; ESRD, end-stage renal disease; IQR, interquartile range; MV, mitral valve; NYHA, New York Heart Association; PMR, primary mitral regurgitation; SMR, secondary mitral regurgitation; STS, Society of Thoracic Surgeons.

### Hemodynamic Parameters

**Figure 2:**
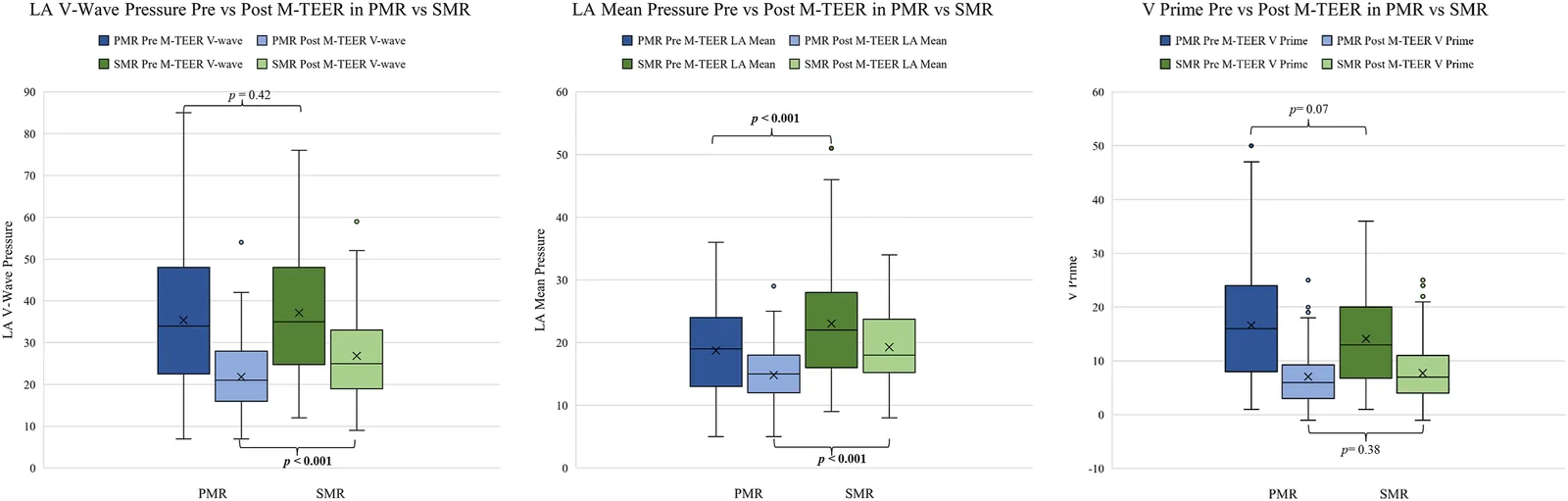
**Pre–and post–M-TEER LA V-wave, mean and V′ pressures in primary vs. secondary MR.** This figure illustrates the difference in LA V wave, LA mean pressure, and LA V′ between PMR and SMR before and after M-TEER, displayed as mean ± SD and compared using an independent sample *t*-test Abbreviations: LA, left atrial; MR, mitral regurgitation; M-TEER, mitral transcatheter edge-to-edge repair; PMR, primary mitral regurgitation; SMR, secondary mitral regurgitation; V′, V prime.

The overall population showed a significant reduction in LVEF, LA V-wave, LA mean, and V′ pressures after M-TEER. Patients with SMR had higher pre–M-TEER LA mean pressures than patients with PMR (23.19 ± 8.49 vs. 18.79 ± 7.08; *p* < 0.001) (Figure 2). Post M-TEER, SMR patients had higher LA V-wave and mean pressures compared to PMR patients (26.84 ± 10.31 vs. 21.82 ± 8.53; *p* < 0.001) (19.29 ± 6.07 vs. 14.88 ± 4.54; *p* < 0.001), respectively (Table 2) (Figure 2). Both groups, though, showed a significant decrease in LA V-wave and mean pressures after M-TEER. The V′ also decreased significantly after M-TEER in patients with both PMR and SMR (Table 2). Interestingly, V′ before and after M-TEER did not significantly differ between PMR and SMR (Figure 2).

**Table 2:** Pre– and post–M-TEER invasive hemodynamic parameters

	Overall			PMR			SMR		
	Pre–M-TEER	Post–M-TEER	*P* value	Pre–M-TEER	Post–M-TEER	*P* value	Pre–M-TEER	Post–M-TEER	*P* value
LVEF, %	50.39 ± 14.42	48.14 ± 14.11	**<0.001**	57.54 ± 9.14	54.56 ± 8.93	**<0.001**	37.68 ± 13.34	36.72 ± 14.46	0.27
V-wave pressure, mm Hg	36.00 ± 16.33	23.60 ± 9.49	**<0.001**	35.37 ± 16.63	21.82 ± 8.53	**<0.001**	37.15 ± 15.80	26.84 ± 10.31	**<0.001**
LA mean pressure, mm Hg	20.36 ± 7.88	16.45 ± 5.54	**<0.001**	18.79 ± 7.08	14.88 ± 4.54	**<0.001**	23.19 ± 8.49	19.29 ± 6.07	**<0.001**
SBP, mm Hg	110.14 ± 18.71	111.95 ± 17.05	0.21	110.50 ± 18.99	114.00 ± 18.06	0.08	109.37 ± 18.27	107.68 ± 13.92	0.33
DBP, mm Hg	59.36 ± 11.49	57.42 ± 9.94	**0.01**	58.02 ± 11.03	56.32 ± 9.74	0.09	62.15 ± 12.01	59.72 ± 10.05	**0.04**
V prime, mm Hg	15.86 ± 10.02	7.32 ± 5.18	**<0.001**	16.68 ± 10.64	7.08 ± 4.85	**<0.001**	14.36 ± 8.67	7.74 ± 5.75	**<0.001**

Continuous variables are represented as mean ± SD.

Abbreviations: DBP, diastolic blood pressure; LA, left atrial; LVEF, left ventricular ejection fraction; M-TEER, mitral transcatheter edge-to-edge repair; PMR, primary mitral regurgitation; SBP, systolic blood pressure; SMR, secondary mitral regurgitation; V prime, V wave pressure minus LA mean pressure.

In the overall cohort, V′ preprocedurally and postprocedurally was lower in patients with ≤ 2+ residual MR following M-TEER. The change in S/D ratio of PV flow was lower in the group that had >2+ residual MR after M-TEER (Table 3). In Table 4, we found that 43% of patients who had post–M-TEER V′ ≤13 mmHg had a V-wave larger than 22 mmHg. None of the patients who had a post–M-TEER V′ >13 mmHg had a V-wave pressure that was ≤22 mmHg (Table 4).

**Table 3:** Changes in invasive hemodynamic parameters in patients with higher vs. lower mitral regurgitation after M-TEER

	Overall			PMR			SMR		
	Post–M-TEER MR ≤2+	Post–M-TEER MR >2+	*p* value	Post–M-TEER MR ≤2+	Post–M-TEER MR >2+	*p* value	Post–M-TEER MR ≤2+	Post–M-TEER MR >2+	*p* value
Δ V	−11.95 ± 13.95	−16.44 ± 16.92	0.23	−12.91 ± 14.80	−18.88 ± 15.31	0.16	−10.25 ± 12.22	−10.86 ± 20.29	0.94
Pre–M-TEER V wave	34.92 ± 16.15	45.74 ± 14.93	**0.003**	34.16 ± 16.48	45.44 ± 14.67	**0.01**	36.28 ± 15.55	46.43 ± 16.68	0.17
Post–M-TEER V wave	22.971 ± 9.06	29.304 ± 11.45	**0.02**	21.25 ± 8.06	26.56 ± 10.91	0.08	26.03 ± 9.95	35.57 ± 10.81	0.06
ΔV prime	−8.20 ± 8.63	−11.73 ± 10.89	0.15	−9.22 ± 9.41	−12.87 ± 9.86	0.19	−6.37 ± 6.72	−9.29 ± 13.35	0.59
Pre–M-TEER V prime	15.04 ± 9.84	22.14 ± 10.05	**0.004**	15.89 ± 10.48	23.00 ± 10.30	**0.02**	13.53 ± 8.44	20.29 ± 9.99	0.13
Post–M-TEER V prime	6.98 ± 4.89	10.22 ± 6.60	**0.004**	6.73 ± 4.52	9.88 ± 6.38	0.07	7.43 ± 5.51	11.00 ± 7.55	0.26
Δ S/D ratio	1.27 ± 0.98	0.35 ± 0.86	**< 0.001**	1.33 ± 0.93	0.39 ± 0.64	**< 0.001**	1.16 ± 1.06	0.25 ± 1.29	0.15
Δ E	0.12 ± 0.34	0.11 ± 0.41	0.97	0.05 ± 0.34	0.04 ± 0.36	0.93	0.22 ± 0.32	0.28 ± 0.50	0.74

Values are mean ± SD.

Abbreviations: MR, mitral regurgitation; M-TEER, mitral transcatheter edge-to-edge repair; PMR, primary mitral regurgitation; SMR, secondary mitral regurgitation.

**Table 4:** Post-mitral TEER LA V-wave pressure according to post-mitral TEER V′ cutoff of 13 mmHg

	Post–M-TEER V′ ≤13 mmHg, N (%)	Post–M-TEER V′ >13 mmHg, N (%)	*p* value
Post–M-TEER V-wave ≤22 mmHg∗	113 (57)	0 (0)	**<0.001**
Post–M-TEER V-wave >22 mmHg∗	87 (43)	26 (100)	

Categorical variables are represented as N (%).

Abbreviations: LA, left atrial; M-TEER, mitral transcatheter edge-to-edge repair.

∗ 22 mmHg was the median V-wave pressure of the cohort.

**Figure 3:**
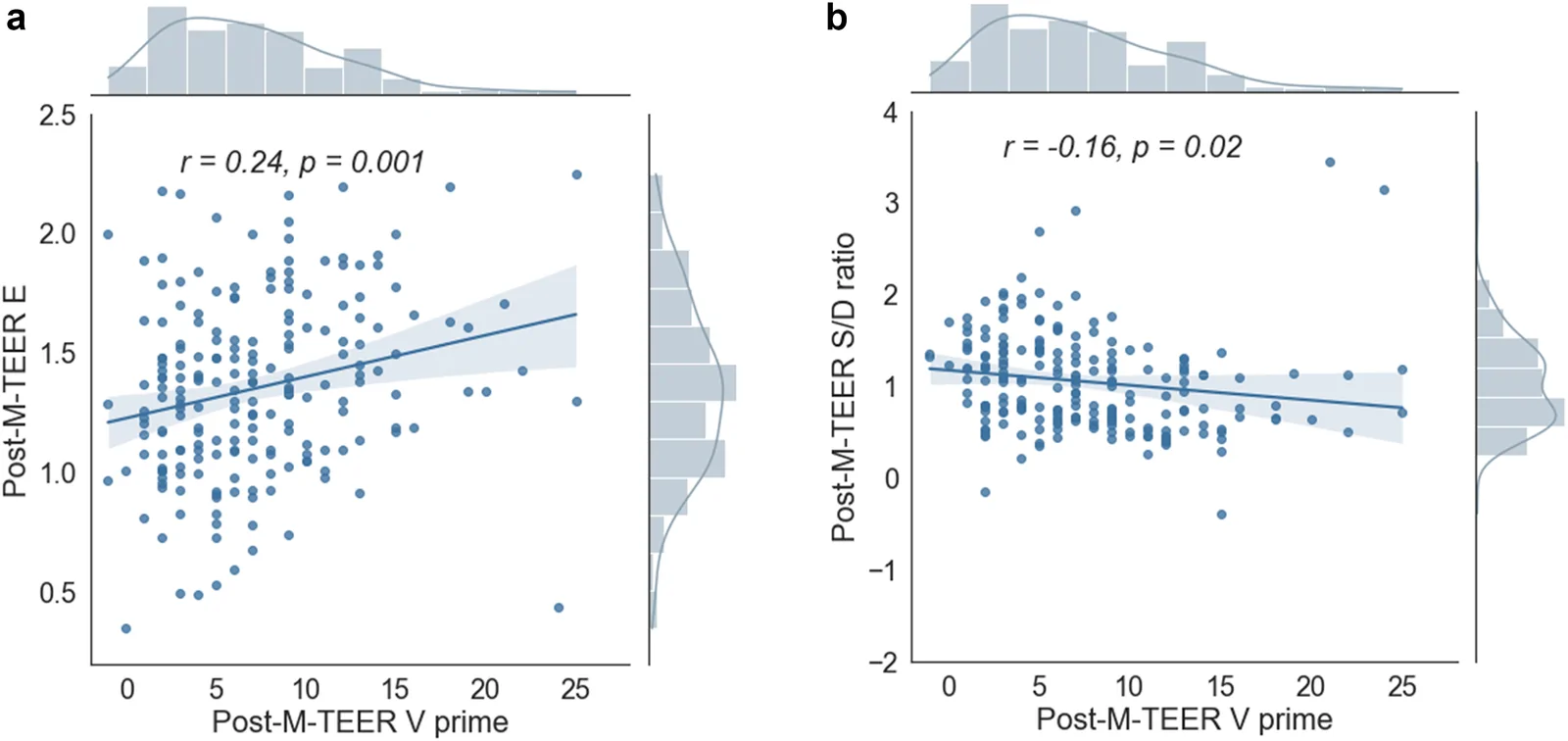
Correlation plots of LA V prime and MV Doppler E wave (A) and LA V prime and PVF S/D ratio (B): Figure (A) illustrates the correlation of post–M-TEER V prime and post–M-TEER E wave on MV Doppler, and Figure (B) illustrates the correlation of post–M-TEER V prime and post–M-TEER S/D ratio of pulmonary venous flow. The correlation was assessed using the Pearson correlation test. Abbreviations: LA, left atrial; M-TEER, mitral transcatheter edge-to-edge repair; MV, mitral valve; PVF, pulmonary vein flow; S/D, systolic/diastolic.

**Figure 4:**
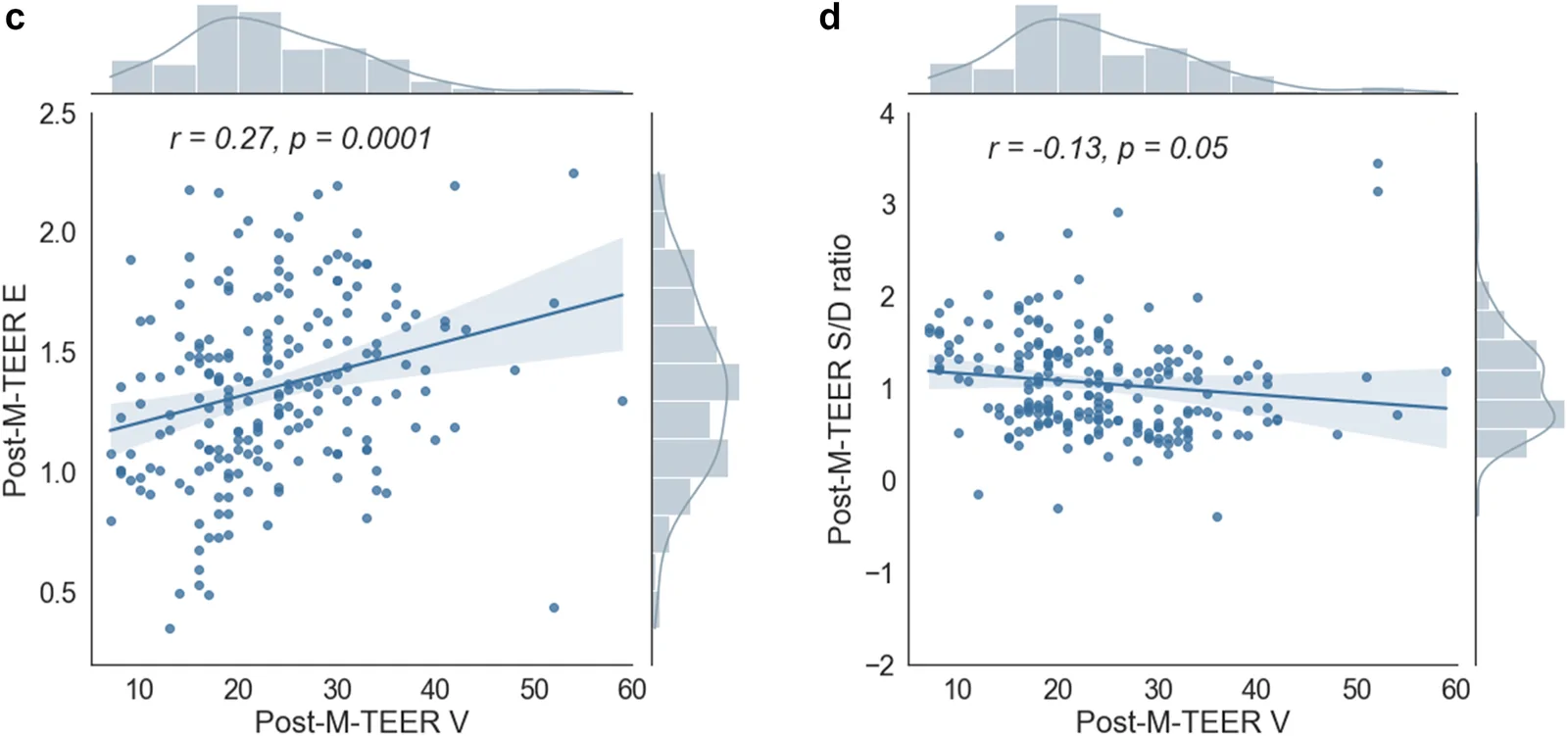
Correlation plots of LA V wave and MV Doppler E wave (C) and LA V wave and PVF S/D ratio (D): Figure (C) illustrates the correlation of post–M-TEER LA V wave and post–M-TEER E wave on MV Doppler, and Figure (D) illustrates the correlation of post–M-TEER LA V wave and post–M-TEER S/D ratio of pulmonary venous flow. The correlation was assessed using the Pearson correlation test. Abbreviations: LA, left atrial; M-TEER, mitral transcatheter edge-to-edge repair; MV, mitral valve; PVF, pulmonary vein flow; S/D, systolic/diastolic.

When assessing the correlation between post–M-TEER V′ and post–M-TEER E wave, we found a positive correlation between those 2 variables (r = 0.24, *p* = 0.0006) (Figure 3A). There was also a negative, yet weak, correlation between post–M-TEER V′ and post–M-TEER S/D ratio (r = −0.16, *p* = 0.02) (Figure 3B). Figure 4 shows a significant positive correlation between post–M-TEER V-wave and post–M-TEER E wave (r = 0.27, *p* < 0.001) (Figure 4C). Interestingly, the negative correlation between post–M-TEER V wave and post–M-TEER S/D ratio was not statistically significant (r = −0.13, *p* = 0.05) (Figure 4D).

### Clinical Outcomes

**Figure 5:**
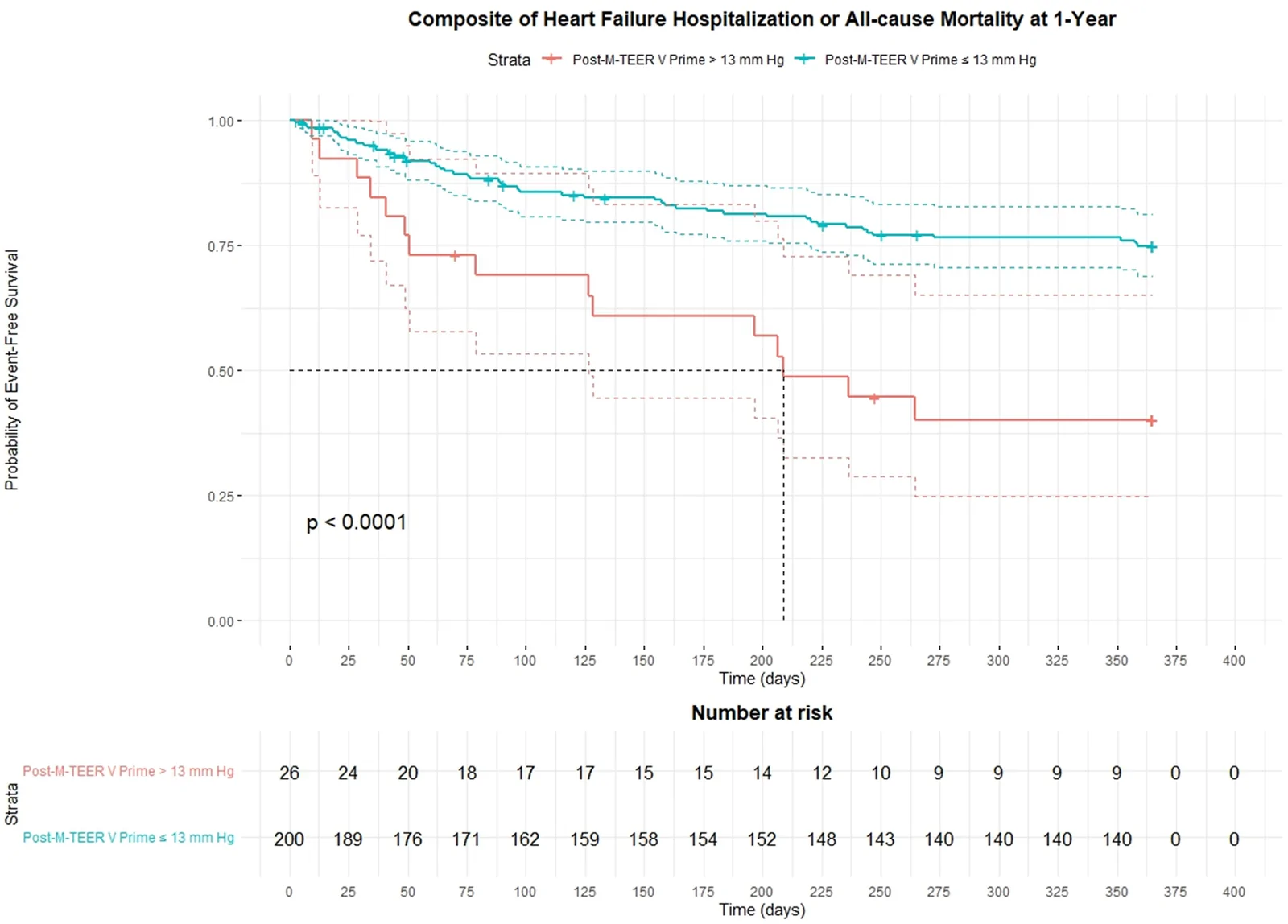
**Kaplan–Meier curve of composite outcome of HFH or all-cause mortality at 1 year.** This Kaplan–Meier curve illustrates the difference in composite outcome of heart failure hospitalization or all-cause mortality at 1 year in patients who underwent M-TEER stratified by their postprocedural V′ pressure of >13 mmHg and V′ ≤13 mmHg. Patients who had a final V′ pressure >13 mmHg showed worse composite outcome compared to patients with V′ ≤13 mmHg when compared using the log-rank test Abbreviations: HFH, heart failure hospitalization; M-TEER, mitral transcatheter edge-to-edge repair; V′, V prime.

Patients who had post-M-TEER V′ >13 mmHg had a higher cumulative probability of a composite outcome of HFH or all-cause mortality at 1 year when compared to patients who had a postprocedural V′ ≤13 mmHg (Figure 5). In the unadjusted Cox regression analysis, patients with post–M-TEER V′ of >13 mmHg had higher hazards for the composite of HFH or all-cause mortality (hazard ratio [HR]: 3.01; 95% CI: 1.68-5.4; *p* < 0.001), all-cause mortality (HR: 3.07; 95% CI: 1.43-6.58; *p* < 0.001), and HFH (HR: 2.51; 95% CI: 1.19-5.26; *p* = 0.02). After adjustment for the etiology of MR and pre–M-TEER V′, patients with post–M-TEER V′ of >13 mmHg had higher hazards of the composite outcome of HFH or all-cause mortality and all-cause mortality (Table 5).

**Table 5:** Unadjusted and adjusted Cox regression analysis for clinical outcomes of patients with higher vs. lower post–M-TEER V′

Outcome	Groups based on post–M-TEER V prime cutoff, mm Hg	No. of events, n (%)	Unadjusted			Adjusted∗		
			HR	CI (95%)	*p* value	HR	CI (95%)	*p* value
Composite of heart failure hospitalization or all-cause mortality	≤13	46 (24)	Reference					
	>13	15 (58)	3.01	1.68, 5.4	**<0.001**	2.74	1.43, 5.25	**<0.001**
All-cause mortality	≤13	25 (13)	Reference					
	>13	9 (35)	3.07	1.43, 6.58	**<0.001**	3.13	1.32, 7.44	**0.01**
Heart failure hospitalization	≤13	32 (17)	Reference					
	>13	9 (35)	2.51	1.19, 5.26	**0.02**	2.3	1.01, 5.22	0.05

Data are hazard ratios (95% CIs).

Bold *p* values are significant at <0.05.

Abbreviations: HR, hazard ratio; M-TEER, mitral transcatheter edge-to-edge repair.

∗ Adjusted for the etiology of mitral regurgitation and pre–M-TEER V Prime.

## Discussion

In our study, we compared baseline and postprocedural invasive LA pressure and hemodynamic parameters between PMR and SMR patients undergoing M-TEER. Our key findings are as follows: V′ decreases significantly after M-TEER irrespective of the mechanism of MR; LA V-wave also decreases after M-TEER in PMR and SMR, but postprocedural V-wave is higher in SMR patients compared to PMR patients; V′ correlates with MV E wave and pulmonary venous flow S/D ratio, although the correlation is modest; V′ is lower in patients with <2+ residual MR (compared to >2+ residual MR) in both PMR and SMR; and patients with post–M-TEER V′ >13 mmHg had worse composite outcomes of HFH or all-cause mortality.

Although the LA V-wave was similar in SMR compared to PMR patients before M-TEER, post–M-TEER, LA V-wave, and LA mean pressures were significantly higher in SMR. This is expected, as LV dysfunction in SMR patients leads to overall higher LA pressures. However, a successful procedure resulted in a similar V′. Our findings corroborate a study that also showed a significant difference in LA V-wave and mean pressures between PMR and SMR after M-TEER.^5^ Another study showed a decrease in the ventricular V-wave of pulmonary capillary wedge pressure (PCWP) after M-TEER but did not stratify based on MR etiology.^3^ In a study with a majority of SMR patients, mean PCWP, PCWP V-wave, LA mean and V-wave pressures, and mean pulmonary artery pressure decreased after M-TEER in their entire cohort.^4^ None of those studies assessed and compared the V′ in PMR and in SMR patients, a measure that we propose as a novel index that better reflects MR severity independent of LV filling.

Elevated LA V-waves are not only found in MR and should be interpreted with caution.^6^^,^^7^ Even in patients with MR, elevation in the amplitude of the LA V-wave is multifactorial, caused by both the regurgitation of blood into the LA (MR) and by LV diastolic dysfunction with varying contributions. Hence, we theorize that the V′ represents the contribution of the MR to the LA V-wave measured above the LA mean pressure, which may explain why V′ may be a better correlate to MR severity than V-wave or LA mean pressure independently. The reduction in LA V-wave and mean pressures seen in PMR and SMR after M-TEER can be attributed to the resolution of MR achieved by the procedure. The higher LA mean and V-wave pressures in SMR patients, who had a lower LVEF compared to PMR patients, support the fact that LV diastolic dysfunction also contributes to the elevation of LA V-wave and mean pressures.^8^, ^9^, ^10^, ^11^ Although patients with PMR may also have cardiomyopathy that can contribute to the elevation of the V-wave, we hypothesize that MR plays a bigger role than diastolic dysfunction in the elevation of the V-wave in PMR patients, and the resolution of MR by M-TEER explains the lower pressures after M-TEER in this group. While in SMR, the higher pressures after M-TEER could be explained by the diastolic dysfunction that is unresolved by the M-TEER procedure. Given the multifactorial nature of the V-wave, discordance of the LA V-wave and MR quantification on echocardiography after placement of M-TEER devices can occur. We hypothesize that V′ can be a valuable intraprocedural parameter to aid in guiding the need for the addition of more M-TEER devices. A noteworthy caveat is that the procedure may also reduce LA mean pressure, which, in the absence of mitral stenosis, correlates with LV pressure. Nonetheless, V′ appears to be a better correlate to MR severity given the more dramatic decrease in V′ following the procedure.

To grade the size of V waves relative to the severity of MR, Fuchs et al.^12^ looked at the height at which the pulmonary capillary wedge V wave exceeded the mean pressure. We used the difference between the V wave and the mean pressure of the LA measured directly from the LA invasively during the M-TEER procedure and named this difference V′. In our study, 43% of patients who had a post–M-TEER V′ ≤13 mmHg had post–M-TEER V-waves that were >22 mmHg, while in patients who had a post-M-TEER V′ >13 mmHg, none had a V-wave pressure ≤22 mmHg; this supports the fact that LA V′ can be a better parameter to assess residual MR than V waves. Our study showed that the V′ decreases after M-TEER in both PMR and SMR, which can be attributed to the resolution of MR and reduction in V-wave and could mean that the M-TEER effect on the MR portion of the LA V-wave is similar in patients with PMR and SMR and is accurately reflected on this novel parameter.

A study by El Shaer et al.^13^ that assessed PV flow morphology after M-TEER as a predictor of survival showed that PV flow blunting or reversal after the procedure was independently associated with mortality. They also showed that those patients were more likely to have higher LA mean and V-wave pressures preprocedurally and postprocedurally and more residual MR after M-TEER. Another study showed that improvement in PV waveform and a greater decrease in V-wave predicted lower hazards for all-cause mortality.^14^ Klein et al.^15^ addressed the effect of MR on pulmonary venous flow and LA pressure. They found that reversed PV flow and smaller S/D ratios reflect a higher LA V wave in patients who had severe MR. Notably, the sample size in that study was limited to 16 patients, which could explain the stronger correlation found between LA V wave and PV S/D ratio. In our analysis, the postprocedural V′ was significantly associated with the postprocedural S/D ratio of PV flow, while the association of the S/D ratio with postprocedural LA V-wave was not significant. Our survival analysis also showed that a postprocedural V′ of >13 mmHg was associated with a more composite outcome of HFH or all-cause mortality. This association was still significant when adjustment for the etiology of MR and the pre–M-TEER V′. Rottländer et al.^16^ demonstrated that patients with post–M-TEER V-waves of less than 25 mmHg had lower 3-year mortality compared with those with post–M-TEER V-waves greater than 25 mmHg. However, this difference in mortality was not observed at 1 year between the 2 groups. In a multivariable analysis, post–M-TEER V-waves >25 mmHg were not predictive of mortality at 1 year, but they were found to be predictive of mortality at 3 years. The lack of a significant prognostic value for V-waves at 1 year was thought to be due to the time required for residual MR or LA/LV restriction to lead to clinical outcomes. The authors proposed that a V-wave of <25 mmHg could serve as a prognostic factor in both functional mitral regurgitation and PMR patients, while degenerative mitral regurgitation patients showed a marked benefit from V-wave reduction. This aligns with our proposed theory that the V wave in itself is not driven purely by MR and is also influenced by LV filling pressures. In our cohort, patients with a V prime >13 mmHg had a higher hazard of the composite outcome of HFH or all-cause mortality and all-cause mortality at 1 year, suggesting V prime as a more accurate measure of residual MR.

There has been an effort to understand hemodynamic changes during M-TEER and to use them to guide decision-making. This study could help us better understand the LA pressure hemodynamic waveform and its change in response to M-TEER therapy and to explore innovative parameters in LA pressure measurement. Multimodality assessment remains crucial, and V′ can be used to complement other intraprocedural data—including echocardiographic measurements, invasive hemodynamic assessment, and clinical context—to guide decision-making. Our findings suggest V′ to be a more specific marker of MR severity, making it a valuable parameter to help guide the need for additional M-TEER devices in the setting of discordance of V wave measurement and MR quantification on echocardiography intraprocedurally and a better predictor of clinical outcomes.

## Limitations

This is a retrospective, single-center study describing M-TEER–induced hemodynamic changes and has the inherent limitations of a retrospective study. Data analysis was performed retrospectively; hence, no causal inference could be made. Data on fluid management, general anesthesia, catecholamine therapy, and rhythm abnormalities like atrial fibrillation could potentially serve as confounders. V′ was not studied with an increase in systemic pressure to understand the impact of increased afterload on MR. The unavailability of invasive hemodynamic data for a portion of our cohort raises the possibility of a selection bias. In addition, the V′ cutoff was derived using a data-driven, maximally selected rank statistics approach, which may be prone to type I error inflation and overestimation of effect size; thus, the threshold is cohort specific and requires external validation. A key limitation to implementing invasive hemodynamic parameters, such as V′, in clinical practice is the difficulty in obtaining accurate intraprocedural data, which may hinder widespread adoption.

## Conclusion

Although M-TEER decreased LA mean, V-wave, and V′ pressures in PMR and SMR patients, V′ provides a novel measure that correlates better with clinical outcomes and severity of residual MR compared to these conventional measurements. V′ can be potentially used as a novel measure to help guide the need for additional M-TEER devices intraprocedurally in the setting of hemodynamic and echocardiographic discordance.

## Ethics Statement

The research reported has adhered to the relevant ethical guidelines.

## Disclosure Statement

The authors report no conflict of interest.
