# Supplementary material for: A Novel Invasive Hemodynamic Parameter (V′) to Predict Hemodynamic Response to Mitral Transcatheter Edge-to-Edge Repair
Source: Struct Heart. 2026 Jun 9;10(9):101069. doi: 10.1016/j.shj.2026.101069 (PMC13571545; doi:10.1016/j.shj.2026.101069)
Supplement: Supplementary file 1 [file mmc1.pdf]

Supplementary Table 1: Unadjusted and Adjusted Cox Regression Analysis for Clinical Outcomes of Patients with Higher vs Lower Post-M-TEER V'

Outcome	Groups based on Post-M-TEER V Prime cut-off, mm Hg	No. of events, n (%)	Unadjusted			Adjusted*		
			**HR**	**CI (95%)**	***P*-value**	**HR**	**CI (95%)**	***P*-value**
Composite of Heart Failure Hospitalization or All-cause mortality	≤ 13	46 (24)	Reference					
	> 13	15 (58)	3.01	1.68, 5.4	**<0.001**	2.16	0.99 -4.69	**0.05**
All-cause mortality	≤ 13	25 (13)	Reference					
	> 13	9 (35)	3.07	1.43, 6.58	**<0.001**	2.06	0.65-6.46	0.22
Heart Failure Hospitalization	≤ 13	32 (17)	Reference					
	> 13	9 (35)	2.51	1.19, 5.26	**0.02**	2.16	0.91-5.14	0.08

Data are hazard ratios (95% confidence intervals).
Bold p-values are significant at <0.05.
M-TEER: Mitral Transcatheter Edge-To-Edge Repair
*Adjusted for the aetiology of mitral regurgitation and pre-M-TEER V Prime, Age, DM, BMI, STS MV repair, NYHA
